# Autologous cellular therapy for cerebral palsy: a randomized, crossover trial

**DOI:** 10.1093/braincomms/fcac131

**Published:** 2022-05-20

**Authors:** Charles S. Cox, Jenifer Juranek, Steven Kosmach, Claudia Pedroza, Nivedita Thakur, Allison Dempsey, Kimberly Rennie, Michael C. Scott, Margaret Jackson, Akshita Kumar, Benjamin Aertker, Henry Caplan, Fabio Triolo, Sean I. Savitz

**Affiliations:** Department of Pediatric Surgery, McGovern Medical School at The University of Texas Health Science Center at Houston (UTHealth), Houston, TX, USA; Program in Pediatric Regenerative Medicine, McGovern Medical School at The University of Texas Health Science Center at Houston (UTHealth), Houston, TX, USA; Department of Pediatric Surgery, McGovern Medical School at The University of Texas Health Science Center at Houston (UTHealth), Houston, TX, USA; Program in Pediatric Regenerative Medicine, McGovern Medical School at The University of Texas Health Science Center at Houston (UTHealth), Houston, TX, USA; Department of Pediatric Surgery, McGovern Medical School at The University of Texas Health Science Center at Houston (UTHealth), Houston, TX, USA; Department of Pediatrics, McGovern Medical School at The University of Texas Health Science Center at Houston (UTHealth), Houston, TX, USA; Department of Pediatrics, McGovern Medical School at The University of Texas Health Science Center at Houston (UTHealth), Houston, TX, USA; Department of Pediatrics, McGovern Medical School at The University of Texas Health Science Center at Houston (UTHealth), Houston, TX, USA; Department of Pediatrics, McGovern Medical School at The University of Texas Health Science Center at Houston (UTHealth), Houston, TX, USA; Department of Neuropsychology, NeuroBehavioral Health, Milwaukee, WI, USA; Department of Pediatric Surgery, McGovern Medical School at The University of Texas Health Science Center at Houston (UTHealth), Houston, TX, USA; Department of Surgery, McGovern Medical School at The University of Texas Health Science Center at Houston (UTHealth), Houston, TX, USA; Department of Surgery, McGovern Medical School at The University of Texas Health Science Center at Houston (UTHealth), Houston, TX, USA; Department of Neurology, McGovern Medical School at The University of Texas Health Science Center at Houston (UTHealth), Houston, TX, USA; Department of Surgery, McGovern Medical School at The University of Texas Health Science Center at Houston (UTHealth), Houston, TX, USA; Department of Pediatric Surgery, McGovern Medical School at The University of Texas Health Science Center at Houston (UTHealth), Houston, TX, USA; Program in Pediatric Regenerative Medicine, McGovern Medical School at The University of Texas Health Science Center at Houston (UTHealth), Houston, TX, USA; Department of Neurology, McGovern Medical School at The University of Texas Health Science Center at Houston (UTHealth), Houston, TX, USA

**Keywords:** cerebral palsy, autologous cell therapy, bone marrow mononuclear cells, human umbilical cord cells, magnetic resonance imaging

## Abstract

We examined an autologous mononuclear-cell-therapy-based approach to treat cerebral palsy using autologous umbilical cord blood or bone-marrow-derived mononuclear cells. The primary objective was to determine if autologous cells are safe to administer in children with cerebral palsy. The secondary objectives were to determine if there was improvement in motor function of patients 12 months after infusion using the Gross Motor Function Measure and to evaluate impact of treatment on corticospinal tract microstructure as determined by radial diffusivity measurement. This Phase 1/2a trial was a randomized, blinded, placebo-controlled, crossover study in children aged 2–10 years of age with cerebral palsy enrolled between November 2013 and November 2016. Participants were randomized to 2:1 treatment:placebo. Treatment was either autologous bone-marrow-derived mononuclear cells or autologous umbilical cord blood. All participants who enrolled and completed their baseline visit planned to return for follow-up visits at 6 months, 12 months and 24 months after the baseline visit. At the 12-month post-treatment visit, participants who originally received the placebo received either bone-marrow-derived mononuclear cell or umbilical cord blood treatment. Twenty participants were included; 7 initially randomized to placebo, and 13 randomized to treatment. Five participants randomized to placebo received bone-marrow-derived mononuclear cells, and 2 received umbilical cord blood at the 12-month visit. None of the participants experienced adverse events related to the stem cell infusion. Cell infusion at the doses used in our study did not dramatically alter motor function. We observed concordant bilateral changes in radial diffusivity in 10 of 15 cases where each corticospinal tract could be reconstructed in each hemisphere. In 60% of these cases (6/10), concordant decreases in bilateral corticospinal tract radial diffusivity occurred post-treatment. In addition, 100% of unilateral corticospinal tract cases (3/3) exhibited decreased corticospinal tract radial diffusivity post-treatment. In our discordant cases (*n* = 5), directionality of changes in corticospinal tract radial diffusivity appeared to coincide with handedness. There was a significant improvement in corticospinal tract radial diffusivity that appears related to handedness. Connectivity strength increased in either or both pathways (corticio-striatal and thalamo-cortical) in each participant at 12 months post-treatment. These data suggest that both stem cell infusions are safe. There may be an improvement in myelination in some groups of patients that correlate with small improvements in the Gross Motor Function Measure scales. A larger autologous cord blood trial is impractical at current rates of blood banking. Either increased private banking or matched units would be required to perform a larger-scale trial.

## Teaser

Cox *et al* examined using autologous cell therapy to treat cerebral palsy, comparing autologous umbilical cord blood/bone-marrow-derived mononuclear cells versus placebo. Infusions were safe. There may be improvement in corticospinal tract radial diffusivity and connectivity, but no improvements in motor function (Gross Motor Function Measure scale) in treated patients over controls were observed.

## Graphical Abstract

**Graphical abstract:**
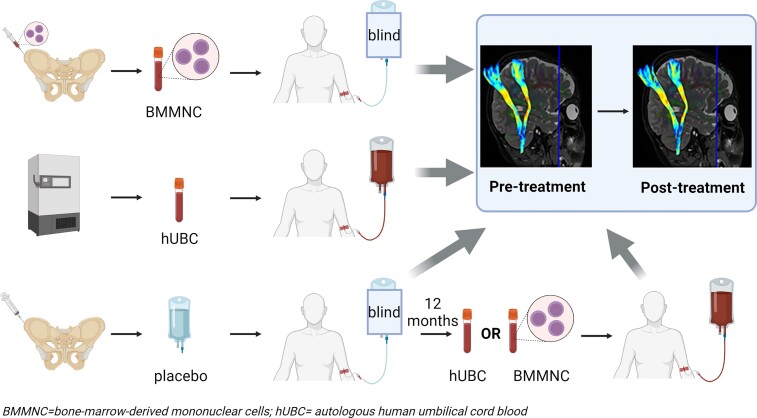

## Introduction

Cerebral palsy (CP) is a phenotypically heterogeneous motor disorder of childhood resulting from a range of CNS insults that can occur in the perinatal period, including *in utero* stroke, hypoxia/ischaemia, and intraventricular/intraparenchymal bleeding.^1^ Children with CP have varying degrees of functional motor deficits that impact the development of gross and fine motor skills, limiting their mobility and ambulation. Current treatments focus on rehabilitative strategies to minimize the impact of spasticity, as well as orthopaedic and neurosurgical procedures.^2^

Currently, there are no curative treatments for CP that restore damaged cortical or corticospinal tracts (CSTs).^2^ However, cell-based therapies using a wide range of stem cells/progenitor cells/monocytes (cord blood mononuclear cells, mesenchymal stem/stromal cells, bone marrow mononuclear cells, neural progenitor cells, M2 macrophages) have been applied in clinical trials to treat various types of CNS injuries, including CP, with the aim of improving neurological function. These approaches have used various delivery modes, including intrathecal, intra-arterial, intramuscular, and intravenous routes.^3–9^

The unifying theme of all of these adoptive cellular therapeutics is the modulation of the dysregulated (acute or chronic) inflammatory response, mostly mediated by microglia.^10–13^ Numerous preclinical investigators have shown that most of these cell types are efficacious in the setting of acute neurological injury/ischaemia.^13–17^ Furthermore, there has been evidence to suggest that specific sub-populations of cord blood contain the biologically active cells such as monocytes responsible for the treatment effect,^18,19^ or regulatory B cells in other cell preparations.^20^

Our study examined an autologous mononuclear cell therapy approach to treat CP in children using autologous human umbilical cord blood (hUCB) if available or bone-marrow-derived mononuclear cells (BMMNCs) if no autologous hUCB was available. The primary objective was to determine if autologous cells are safe to administer in children with CP. The secondary objectives were to determine if there was an improvement in the motor function of patients 12 months after infusion using the Gross Motor Function Measure (GMFM) and to evaluate the impact of treatment on CST microstructure as determined by radial diffusivity (RD) measurement, a commonly used metric to assess myelination of major white matter tracts.^21–23^ Exploratory objectives were related to a wide battery of neurocognitive and functional outcome measures, as the GMFM scale is heavily weighted towards ambulation. In addition, changes in structural connectivity of prominent motor regions were explored using network-based connectivity matrix analyses.

## Participants and methods

Institutional Review Board approval was obtained from the Committee for the Protection of Human Subjects at UTHealth and the Memorial Hermann Hospital System (HSC-MS-12-0876). Written informed consent was obtained from either 1 or both parents of the participants and assent from the child, if possible, prior to enrolment. This study was conducted in accordance with the tenets of the Declaration of Helsinki, Health Insurance Portability and Accountability Act, and with an assurance filed with and approved by the Food and Drug Administration (Federal Investigational New Drug Application 15246). This study was registered with ClinicalTrials.gov (NCT01988584).

### Trial design

**Figure 1:**
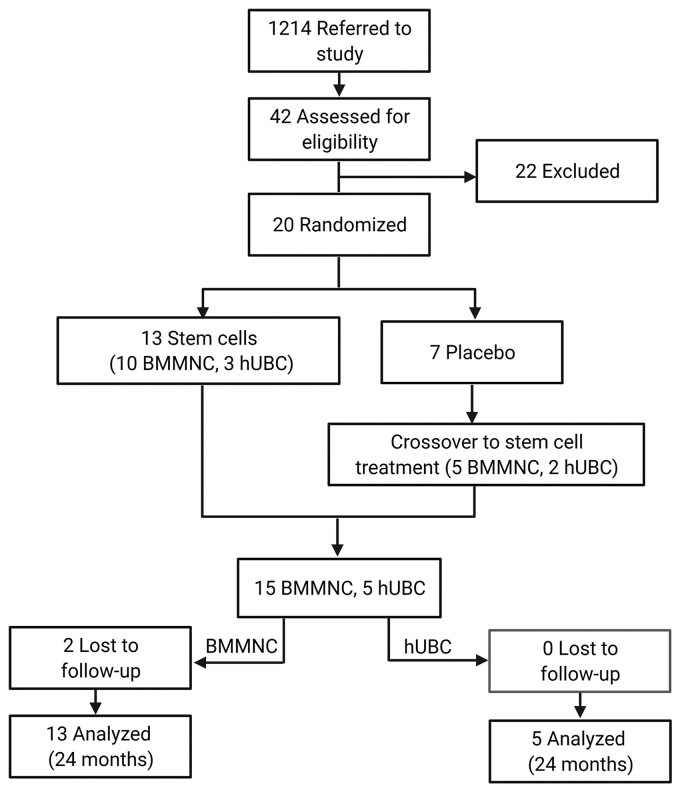
**CONSORT diagram.** hUCB = human umbilical cord blood; BMMNC = bone marrow mononuclear cells. Created with BioRender.com.

This Phase 1/2a trial was a randomized, blinded, placebo-controlled, crossover study primarily designed to investigate the safety and secondarily the efficacy of intravenous (IV) infusion of autologous hUCB or BMMNC treatment in children between 2 and 10 years of age with CP. A total of 30 participants (15/arm) were planned to be enrolled in the study. Participants were randomized to 2:1 treatment:placebo. Treatment was either autologous BMMNC or autologous hUCB if available. All participants who enrolled and completed their baseline visit planned to return for follow-up visits at 6 months, 12 months and 24 months after the baseline visit. At the 12-month post-treatment visit, participants who originally received the placebo received either BMMNC or hUCB treatment (Fig. 1). All study visits and procedures were conducted at The University of Texas Health Science Center at Houston (UTHealth) Pediatric Surgery Clinic and Children’s Memorial Hermann Hospital in Houston, TX.

### Objectives and outcomes

The primary objective of this study was to determine if autologous cells using either BMMNCs or hUCBs are safe to administer in children with CP. Primary safety outcome measures were in-hospital toxicity (pulmonary and hepatic function; new seizures, haemorrhagic lesions, or ischaemic lesions on imaging) and long-term safety measures (development of new mass lesions or other pathological structural changes; worsening neurological status). The secondary objective was to determine if structural and/or functional outcomes were improved following administration of autologous cells compared with participants in the control condition. Secondarily, efficacy of treatment with autologous cells was evaluated longitudinally with outcome measures which included quantitative structural neuroimaging metrics from diffusion MRI (dMRI) as well as standardized functional assessments of motor skills (GMFM-88) and adaptive behaviour (Vineland Adaptive Behavior Scales—Second Edition [VABS-2]). Functional outcomes at baseline and 12 months post-treatment were compared.

### Randomization

Randomization was computer-generated for treatment versus placebo participants. If participants had hUCB available, they received that treatment. Participants were assigned to the treatment or placebo arms in a 2:1 ratio using sequentially numbered, opaque, sealed envelopes prepared by a member of the UTHealth Center for Surgical Trials and Evidence-based Practice not involved in the clinical trial. Participants, parents, care providers, and primary outcome observers were blinded to treatment. Eligibility criteria (inclusion/exclusion) are listed in Supplemental Table 1.

### Recruitment and screening process

Participants were enrolled between November 2013 and November 2016. Self-referrals from ClincalTrials.gov generated the most inquires, with referrals from the Cord Blood Registry (CBR; CBR Systems, Inc.) also providing participants. A screening telephone call was completed with each potential participant to assess basic study eligibility. Parents who submitted a signed copy of the informed consent along with their child’s medical records, including brain CT and MRI images, were considered in the order received. The principal investigator (C.S.C.) and neurologist co-investigator (S.I.S.) reviewed each child’s medical history and brain CT/MRI images to ensure eligibility. Eligible participants were then scheduled for treatment.

### Intervention

Each participant received a single dose of IV-administered autologous BMMNC or hUCB or placebo (0.9% saline with 5% v/v human serum albumin for BMMNC group and dextran 40 with 5% v/v human serum albumin for hUCB group) over 15 min. For participants receiving BMMNC, BMMNCs were harvested as previously described.^24^ Harvest was done just prior to the baseline imaging study under a combination of local anaesthesia (1% lidocaine) while sedated for the imaging study. No patients exhibited any discomfort with the procedure. A dose of 6 × 10^6 ^cells/kg body weight was infused over 15 min at a 1 million cells/ml concentration. This dose was determined from the maximal reasonable cell number obtainable from a bone marrow harvest of 3–5 ml/kg body weight; this harvest volume has proven safe and haemodynamically benign.^24^ In addition, preclinical data from acute studies have shown a dose of 2–10 × 10^6^ cells/kg was efficacious in stroke and traumatic brain injury rodent models.^14,24,25^

For participants receiving hUCB, previously banked hUCB was obtained from CBR. Umbilical cord blood was collected at the time of birth from consenting mothers who elected to preserve cord blood for their own family’s use and transported to CBR for processing and long-term storage. Upon receipt at CBR, collection volume, transit time, cell viability, and total nucleated cell (TNC) counts (×10^6^ cells) were assessed prior to processing with the automated AXP AutoXpress Platform (ThermoGenesis). If approved for the study, participants’ cryopreserved cord blood unit was transported via medical courier to UTHealth/Children’s Memorial Hermann Hospital for reconstitution prior to infusion. The minimum acceptable dose was 2 × 10^6 ^cells/kg body weight, and the maximum allowable dose was 10 × 10^6 ^cells/kg, with the target dose being the maximum available dose within this range. These doses are in agreement with previously reported effective doses.^26,27^ The bone marrow harvests, hUCB reanimation, cell processing, and product infusions occurred at Children’s Memorial Hermann Hospital and the Evelyn H. Griffin Stem Cell Therapeutics Laboratory at UTHealth. Additional lab processing occurred at the Memorial Hermann Hospital Laboratory. Follow-up was completed in February 2018.

For participants receiving placebo, a sham harvest procedure was performed similarly to the BMMNC harvest procedure.^24^ An 11-gauge or 15-gauge needle was used to puncture through the skin, but it was not inserted into the iliac bone; no bone marrow aspiration or harvest was performed. The puncture site was then steri-stripped closed and covered with an identical external bandage, similar to the BMMNC procedure. Participants received an infusion of placebo after the sham procedure. The sham procedure was only carried out in the BMMNC group as the families knew they were assigned to the hUCB arm of the study. All other procedures were performed identically in all groups to maintain blinding. Clinical care providers were not allowed to observe the harvests and were not informed of the participants’ assigned treatment groups.

All participants received post-infusion monitoring for harvest/infusion-related toxicities (pulmonary, hepatic, and renal lab indices) and neurologic complications overnight in the paediatric observation unit. Participants in the bone marrow harvest group were also monitored for post-harvest puncture site infections. Participants had routine lab work before the infusion and the following day before discharge. The 21-day post-infusion summaries for each participant were reviewed by the medical safety monitor.

### Behavioural and motor function assessments

#### Motor functioning

The GMFM-88 is an 88-item standardized, observation-based assessment of children’s gross and fine motor abilities.^28–30^ It was designed for motor assessment of children with CP and takes 45–60 min. Assessments were conducted at baseline and 12 months post-treatment. The same clinician, who was blind to treatment, conducted each motor assessment. Although scoring occurred in real-time, per instrument instructions, each assessment was also video recorded for quality and scoring checks. The GMFM-88 scores were converted to GMFM-66 scores after all data were collected using the scoring conversion algorithm software offered on the instrument publisher’s website. The assessor was blinded to treatment.

#### Adaptive behaviour functioning

The VABS-2 is an interview-based parent/caregiver report measure that provides information regarding a child’s adaptive behaviour/daily living skills across multiple domains, including fine and gross motor skills.^31^ The instrument yields both raw scores and standardized scores based on a child’s age. The VABS-2 is appropriate for children from birth through adolescence and produces scores in the following domains: Communication, Daily Living Skills, Socialization, and Motor Skills. The measure takes between 25 and 90 min to complete and was administered by clinicians with backgrounds in psychological assessment who were blinded to treatment. Assessments were conducted at baseline and 12 months post-treatment.

### Neuroimaging

#### Acquisition

The same 3T Signa LX (8-channel head coil) clinical scanner (GE Healthcare) located at Children’s Memorial Hermann Hospital in Houston, TX, was used for acquiring all longitudinal MRI data throughout the study period (2013–2018). dMRI and structural MRI were acquired at 3 time points: baseline (prior to infusion), 12-month and 24-month study visits. The following 3D sequences were acquired in the sagittal plane: T_1_-weighted spoiled gradient echo (SPGR; 1 mm^3^ isotropic voxel dimensions; repetition time [TR]: 6.98 ms; time to echo [TE]: 2.84 ms; flip angle: 11°); T_2_-weighted fast spin echo (1 mm^3^ isotropic voxel dimensions; TR: 3000 ms; TE: 71.24 ms; flip angle: 90°); and T_2_-weighted fluid-attenuated inversion recovery (FLAIR; voxel dimensions: 0.57 × 0.57 × 1 mm^3^; TR: 6000 ms; TE: 117 ms; inversion time [TI]: 1883 ms; flip angle: 90°). In addition, the following scans were acquired in the axial plane: 3D susceptibility-weighted angiography (SWAN; voxel dimensions: 0.47 × 0.47 × 1.5 mm^3^; TR: 40.7 ms; TE: 25.2 ms; flip angle: 20°) and 30 direction^32^ Echo-planar imaging-based spin echo dMRI sequence (Jones 30 scheme; 2.7 mm^3^ isotropic voxel dimensions; TR: 14 900 ms; TE: 82.3 ms; b = 1000 s/mm^2^) for diffusion tensor fitting and reconstruction of common scalar maps for each of the following diffusion tensor imaging (DTI) metrics: fractional anisotropy (FA), mean diffusivity (MD), RD, and axial diffusivity (AD). Completing the entire multimodal protocol required 65 min of imaging time per imaging session in each participant.

### Analyses

#### Corticospinal tract metrics (changes in radial diffusivity, a quantitative marker of myelination)

**Figure 2:**
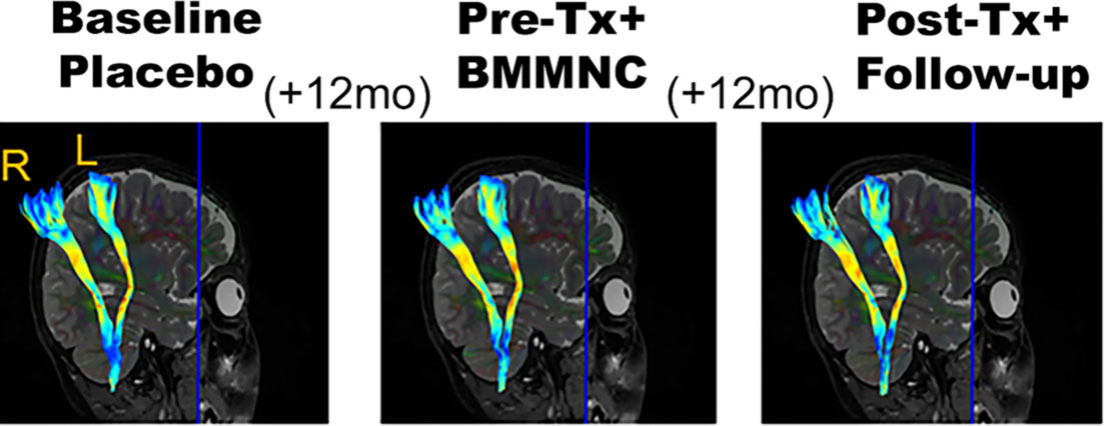
**Longitudinal cortical spinal tract reconstruction (bilaterally) in a single patient.** Right and left hemisphere CSTs are labeled in first panel on the left. This particular patient was initially randomly assigned to the ‘placebo first’ condition and then crossed-over to the treatment (TX+) condition 12 months later (BMMNC since no autologous hUCBs were available). The final panel on the right displays CST reconstruction in each hemisphere at 12 months post-treatment with BMMNCs. Hot colours indicate high RD values and cold colours represent low RD values. mo = months

All scans were analyzed blind to diagnosis, treatment, age, and sex. White matter tracts of interest included the left and right hemisphere CSTs (CST_LH and CST_RH, respectively) and were reconstructed using software developed by Alexander Leemans.^33^ Based on the diffusion tensor model, deterministic tractography methods were utilized to reconstruct the CST in each hemisphere in each participant using mask-based seeding in each cerebral peduncle and retaining tracts terminating in each motor cortex. Owing to the longitudinal study design, within-participant co-registration of dMRI data sets to the same T2-weighted image (second time point) was performed to create participant-specific seed masks for tractography of each CST across all time points of dMRI. Each participant’s seed mask in each cerebral peduncle from the 12-month visit was transformed to the same participant’s native diffusion space for baseline and 24-month visits for tractography. Thus, the size and location of each seed mask used for CST reconstruction were constant across all time points within each participant (Fig. 2). A common metric of white matter myelination, RD, was extracted from each CST reconstruction from each imaging session.

#### Network-based structural connective metrics (changes in number of streamlines)

Structural connectivity analyses were conducted using a multimodal approach. Specifically, Freesurfer v6^34^ was implemented to parcellate the cerebral cortex into individual gyri based on delimiting sulci according to the Desikan atlas using the 3D T_1_-weighted sequences. The resulting 34 cortical parcels per hemisphere as well as 6 subcortical segmentations (thalamus, caudate, putamen, globus pallidum, hippocampus and amygdala) were subsequently co-registered with each participant’s eddy-corrected dMRI sequence and used for probabilistic tractography (probtrackx2) to create a structural connectivity matrix using FSL v6.0.1^35^ for each individual participant at each timepoint. Computational resources on a high-performance computing cluster, including graphics processing units (Texas Academic Computing Center, The University of Texas at Austin) accelerated the intensive computing necessary to conduct these analyses.^36–38^

The resultant structural connectivity matrix for each dataset (FSL’s fdt_network_matrix file) was normalized by each respective waytotal file containing the total number of streamlines from each seed mask to create a new corrected matrix that reflected the probability of structural connectivity between pairs of parcels and subcortical structures. Finally, all individual matrices were scaled to the group average to create *z*-transformed values for each data set.

Based on our study hypothesis that IV treatment (with autologous BMMNCs or hUCB) would improve white matter integrity via myelin remodeling, we targeted our analyses to evaluate changes in diffusivity metrics, specifically RD. Since seminal animal model studies by Song *et al*.,^22^ increased RD values have been consistently associated with reduced myelination in translational studies of demyelinating disease and its progression.^23^ In our present study of CP, we hypothesized that (i) CST RD would decrease bilaterally in response to treatment and (ii) changes in CST RD would correlate with changes in behavioural measures of motor function.

#### Sample size and statistical analysis

A total of 30 participants (15/arm) were planned to be enrolled in the study. Thirty participants would provide a preliminary assessment of potential serious adverse events with a 10–15% incidence.^24,39^

Primary analyses were intent-to-treat comparing outcomes between cell therapy (BMMNC and hUCB) treatment and placebo conditions. Imaging variables were analyzed with linear mixed models, including age, time, group, and time-group interaction. We also evaluated a quadratic term for time for each imaging variable. Random intercepts and slopes were included in all models to account for within-patient correlation. Neurocognitive variables were analyzed using the same methods to evaluate longitudinal changes and group differences. Secondary analyses compared outcomes for all participants at pre-infusion to 12 months post-infusion. Mean change scores (12 months post-infusion minus baseline score) and standardized response means were calculated for neurocognitive outcomes to compare to minimal clinically important differences (MCIDs). For GMFM-66 scores, we calculated predicted 12-month scores based on published percentiles.^28^ We then calculated the difference between observed and predicted GMFM-66 scores. All analyses were conducted in R software version 3.5.2. (R Foundation for Statistical Computing).^40^

## Data availability

The data that support the findings from this study are available from the corresponding author upon reasonable request.

## Results

### Participants

A total of 1214 patients were referred for the study: 1200 self-referrals (e.g. ClinicalTrials.gov) and 14 from CBR. Of these, 42 patients were determined to be potentially eligible for the study and were screened. Of those screened, 22 were excluded due to lack of imaging findings to support a diagnosis of CP. However, 20 met eligibility criteria and were enrolled. Treatment either occurred at baseline or the 12-month visit to permit at least 1 follow-up visit 12 months later. Seven participants were initially randomized to the placebo condition, whereas 13 were randomized to treatment. Subsequently, 5 participants initially randomized to the placebo condition at baseline went on to receive BMMNC, and 2 participants received hUCB at the 12-month visit. Of the treatment group, 10 participants received BMMNC, and 3 participants received hUCB. Ultimately, 15 participants received BMMNC, and 5 participants received hUCB. In the BMMNC group, 2 were lost to follow-up before the final visit, which resulted in 13 participants in the BMMNC group and 5 participants in the hUCB group at the 24-month visit (Fig. 1). Twenty participants were enrolled instead of the originally planned 30 participants as there were not any further patients who could be included in the hUCB group with suitable units.

Demographics and baseline participant characteristics are detailed in Supplementary Table 2. There were no significant differences between groups in age and birth characteristics at baseline. The majority of participants (*n* = 9, 45%) were scored as level 5 on the baseline Gross Motor Function Classification System (GMFCS) for CP, with 5 each at levels 3 and 4 (*n* = 10, 50%) and 1 at level 2 (5%). Baseline dystonia movement scale (BDMS) scores and baseline dystonia disability scale (BDDS) scores were not significantly different between groups. Participants had CP characterized as either spastic (*n* = 12, 60%) or dyskinetic (*n* = 8, 40%)^41^ (Supplementary Table 2).

### Safety of bone-marrow-derived mononuclear cells and human umbilical cord blood in children with cerebral palsy

None of the participants experienced adverse events related to the stem cell infusion. No changes were observed in vital signs or clinical lab results of participants between pre- and post-infusion. There was no evidence of in-hospital infusional toxicity, alterations in pulmonary function (chest radiograph and oxygen saturation), alterations in hepatic enzymes, or new imaging findings on follow-up brain axial imaging. The list of adverse events is reported in Supplemental Table 3.

### Behavioural assessments

Behavioural assessment results for all participants and then by treatment condition are detailed in Tables 1 and 2.

**Table 1:** Behavioural assessments at baseline

	Age (y)	GMFM-88	GMFM-66	VABS-2 Comm	VABS-2 Recep	VABS-2 Express	VABS-2 Written	VABS-2 Daily Living	VABS-2 Personal	VABS-2 Domestic	VABS-2 Comm	VABS-2 Social	VABS-2 Motor	VABS-2 ABC
**All groups**														
AVE	5.62	26.37	27.51	63.95	21.00	33.82	5.06	55.89	10.82	2.71	10.35	66.26	45.64	57.74
MEDIAN	5.00	15.55	21.00	61.00	20.00	13.00	0.00	51.00	7.00	0.00	4.00	63.00	45.00	54.00
STDV	2.98	24.83	15.59	20.06	12.54	34.74	8.75	11.65	12.36	4.24	14.06	17.31	15.09	14.37
**BMMNC**														
AVE	6.20	24.23	26.03	63.79	23.67	35.58	6.83	55.21	11.83	2.83	13.33	65.29	43.00	57.64
MEDIAN	7.00	16.83	21.35	60.00	23.00	17.00	3.50	53.00	7.50	0.50	4.50	61.00	45.00	54.00
STDV	3.09	20.62	13.77	19.83	13.26	38.39	9.93	9.59	14.02	4.63	15.88	16.49	11.63	13.56
**hUCB**														
AVE	4.00	32.35	31.66	64.40	14.60	29.60	0.80	57.80	8.40	2.40	3.20	69.00	50.25	58.00
MEDIAN	4.00	7.43	20.50	66.00	12.00	13.00	0.00	50.00	6.00	0.00	2.00	68.00	51.00	54.00
STDV	2.12	36.51	21.16	23.07	8.56	27.24	1.79	17.50	7.77	3.58	2.59	21.27	21.06	18.19

AVE, average; STDV, standard deviation; BMMNC, bone marrow mononuclear cells; hUCB, autologous umbilical cord blood; GMFM-88, Gross Motor Function Measure-88 Scale; GMFM-66, Gross Motor Function Measure-66 Scale; VABS-2, Vineland Adaptive Behavior Scales-Second Edition.

**Table 2:** Behavioural assessments 12-months post-treatment

	Age (y)	GMFM-88	GMFM-66	VABS-2 Comm	VABS-2 Recep	VABS-2 Express	VABS-2 Written	VABS-2 Daily Living	VABS-2 Personal	VABS-2 Domestic	VABS-2 Comm	VABS-2 Social	VABS-2 Motor	VABS-2 ABC
**All groups**														
AVE	6.89	28.38	28.88	62.83	22.53	39.18	7.29	55.44	15.41	4.24	13.88	62.00	44.56	55.06
MEDIAN	7.00	16.08	20.85	61.50	23.00	16.00	5.00	48.00	6.00	1.00	7.00	57.00	43.00	51.00
STDV	2.93	26.04	17.21	19.76	11.72	37.11	9.89	16.51	17.68	5.38	15.39	16.22	13.55	13.85
**BMMNC**														
AVE	7.31	25.92	25.95	62.23	24.33	40.50	8.50	52.54	15.67	4.25	15.75	60.75	40.67	53.58
MEDIAN	8.00	17.31	21.20	61.00	27.50	14.00	3.50	47.00	6.00	1.50	8.50	55.00	41.50	48.00
STDV	3.04	19.83	13.88	20.79	12.88	40.58	11.59	13.20	18.47	5.48	17.18	16.84	11.24	13.78
**hUCB**														
AVE	5.80	34.77	36.5	64.4	18.2	36	4.4	63	14.8	4.2	9.4	65	52.33	58.6
MEDIAN	5.00	7.1	20.5	69	22	18	5	50	6	0	4	63	56	53
STDV	2.59	40.38	24.10	18.96	7.76	31.04	2.61	23.16	17.66	5.76	10.01	16.00	16.80	14.94

AVE, average; STDV, standard deviation; BMMNC, bone marrow mononuclear cells; hUCB, autologous umbilical cord blood; GMFM-88, Gross Motor Function Measure-88 Scale; GMFM-66, Gross Motor Function Measure-66 Scale; VABS, Vineland Adaptive Behavior Scales-Second Edition.

#### Gross Motor Function Measure scores

**Figure 3:**
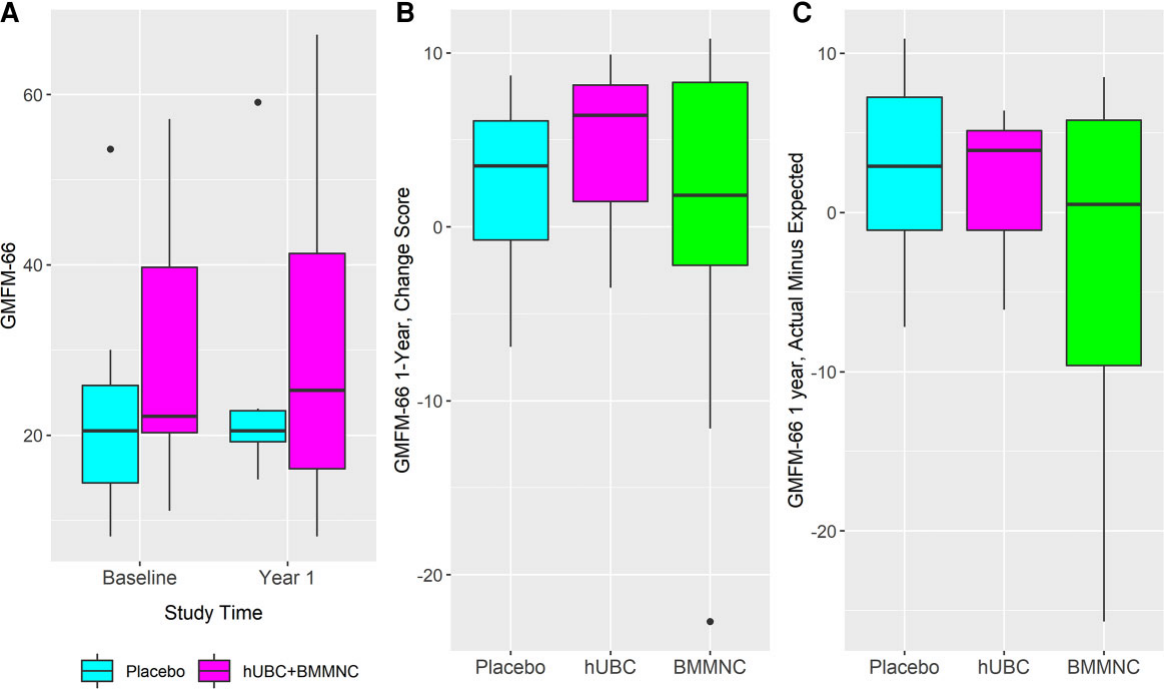
**GMFM-66 Scale Scores.** (**A**) Baseline and 12-month GMFM-66 scores by randomized treatment condition (all cell therapy). Linear mixed model test for interaction between time and group (placebo versus hUCB + BMMNC; *P* value = 0.67). (**B**) Change scores at 12-months post-randomization shown by randomization condition. ANOVA comparing placebo vs hUCB (*P* value = 0.75) and placebo versus BMMNC (*P* value = 0.55). (**C**) Actual minus expected at 12-months post-randomization shown by randomization condition. ANOVA comparing placebo versus hUCB (*P* value = 0.86) and placebo versus BMMNC (*P* value = 0.2).

Figure 3A shows baseline (20 participants) and at 12 months post-treatment GMFM-66 scores by randomized treatment condition (placebo [7] versus all stem cell therapy combined [13]). Figure 3B shows change in scores at 12 months post-treatment by randomization condition. Figure 3C shows the actual minus expected scores at 12 months post-treatment as shown by randomization condition. There were no significant differences between conditions. Based on expected changes in CP in the age range of the study population, for the BMMNC group, 8 participants saw improvement in GMFM scores and 1 remained stable, with no decline noted at 12 months post-treatment. Two of 3 participants, at the third percentile at baseline, had improved GMFM scores at 12 months post-treatment. The remaining participants experienced decline ranging from −0.6 to −3.5.

#### Percentiles

Nine participants in the BMMNC group had improved GMFM-66 scores, 3 were stable (neither improvement nor decline), and 6 showed decline at 12 months. In addition, 2 of the 3 lowest functioning participants showed improvement. This is remarkable because based on GMFM growth curves, these participants would have been expected to decline.

#### Vineland Adaptive Behaviour Scales—Second Edition

With regards to adaptive functioning, mean scores on the motor, communication and social scales were stable at baseline and at 12 months post-treatment. This is notable as patients with adaptive functioning at levels found in our participants typically decline over time. Our BMMNC participants did not show meaningful decline.

### Corticospinal tract imaging

Imaging data were analyzed in a baseline versus 12-months post-treatment manner according to various endophenotypes and handedness (Table 3). Owing to the heterogeneity of clinical phenotypes (the CST was not trackable in 1 side or the other versus both CSTs were trackable) in our CP sample, unilateral CST (*n* = 3) and bilateral CSTs (*n* = 15) endophenotypes (based on gross neuroimaging) were used as an organizational framework for evaluating study results.

**Table 3:** Characteristics of research participants with CP, including change over time in CST RD values relative to intravenous infusion treatment

ID	TX type (BMMNC or hUCB)	Age at Infusion (y)	Handedness	CST reconstruction (Uni or Bi)	TX admin (Tp_1_or Tp_2)	CST RD LH (Txpost-Txpre)	CST RD RH (Txpost-Txpre)	Cerebral Palsy Classification
*Unilateral RD drop*								
16	hUCB	8.7	NA	Uni	Tp_2		−0.018061	Spastic-Quadriplegic
17	hUCB	3	Right	Uni	Tp_1	−0.046882		Hemiparetic
20	hUCB	2.4	Left	Uni	Tp_1		−0.045924	Spastic-Quadriplegic
*Concordant RD drop*								
1	BMMNC	10.9	NA	Bi	Tp_2	−0.005811	−0.013193	Mixed-spastic-Quadriplegic
2	BMMNC	3.8	Left	Bi	Tp_1	−0.021867	−0.018863	Mixed-spastic-Quadriplegic
7	BMMNC	7.7	Left	Bi	Tp_2	−0.00801	−0.031791	Quadriplegic
12	BMMNC	3.2	Right	Bi	Tp_2	−0.061225	−0.013188	Spastic-Dystonic-Quadriplegic
13	BMMNC	2.7	Left	Bi	Tp_1	−0.031761	−0.032738	Quadriplegic
18	hUCB	7.8	NA	Bi	Tp_2	−0.054174	−0.193233	Spastic-Quadriplegic
*Concordant RD increase*								
6	BMMNC	9	Right	Bi	Tp_1	0.05121	0.049008	Quadriplegic
9	BMMNC	3.7	Left	Bi	Tp_1	0.073045	0.038369	Spastic-Quadriplegic
10	BMMNC	10.6	Left	Bi	Tp_1	0.055856	0.127568	Spastic-Diplegic
11	BMMNC	9.1	NA	Bi	Tp_1	0.009728	0.08599	Dyskinetic-Quadriplegic
*Discordant RD changes*								
3	BMMNC	8.5	NA	Bi	Tp_1	−0.032689	0.011876	Spastic-Quadriplegic
4	BMMNC	4.5	Left	Bi	Tp_2	−0.061165	0.053679	Spastic-Diplegic
5	BMMNC	9.5	Left	Bi	Tp_1	−0.03729	0.016587	Dyskinetic-mixed
14	BMMNC	5.1	Right	Bi	Tp_1	0.0111681	−0.011767	Spastic-Diplegic
19	hUCB	4.1	Left	Bi	Tp_1	−0.088097	0.011777	Hemiparetic

CP, cerebral palsy; CST, corticospinal tract; Uni, uilateral; Bi, bilateral; Tp_1, first visit; Tp_2, second visit; RD, radial diffusivity (×10-3 mm^2^/s); TX type, treatment type; TX admin, treatment administration; Txpost, post-treatment; Txpre, pre-treatment (baseline); RH, right hemisphere; LH, left hemisphere; y, year.

#### Longitudinal unilateral corticospinal tract cases (*n* = 3; all hUCB)

**Figure 4:**
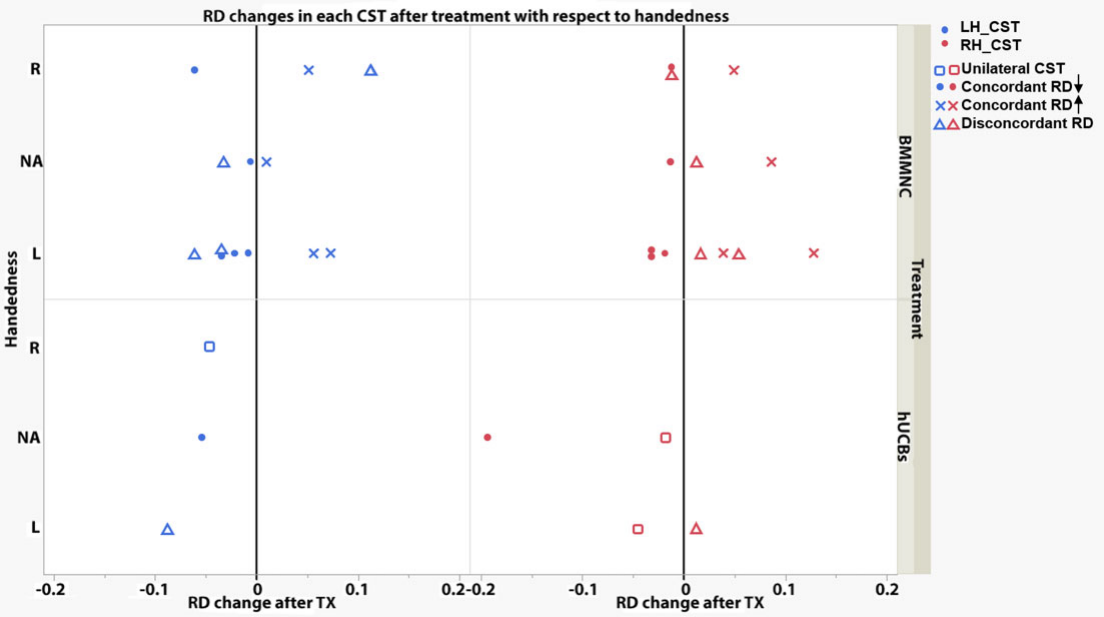
**Quantitative changes in RD values (×10^−3 ^mm^2^/s) in each reconstructed corticospinal tract post-treatment in cerebral palsy participants (*n* = 18).** While negative values indicate RD decreased post-treatment, positive values indicate RD increased post-treatment. Both CSTs (bilaterally; left and right hemisphere) were able to be reconstructed in 15 of 18 participants. For each of these cases, the *directionality* of RD changes was categorized to be either concordant or discordant. For concordant CST changes in RD (*n* = 10), both CST reconstructions reflected either an increase (filled circles; *n* = 4/10) or a decrease (‘x’ symbols; *n* = 6/10) in RD values post-treatment. For discordant cases (triangle symbols; *n* = 5), CST changes in RD occurred in opposite directions. For these discordant cases, handedness appeared to correspond to the hemisphere with decreased CST RD values post-treatment. In the remaining CP cases (3 of 18), only a single CST was able to be reconstructed (square symbols; unilateral CST). In all 3 of these cases, RD values decreased post-treatment. Data presented for individual participants. CST_LH = left hemisphere corticospinal tract; CST_RH = right hemisphere corticospinal tract; TX = treatment.

At 12 months post-treatment, RD significantly decreased in unilaterally reconstructed CST in 3 of 3 cases. A paired samples *t* test showed a significant decrease in RD (t(2) = 3.91, *P* = 0.03, Cohen’s d = 2.26) with an average decrease of 0.037 mm^2^/s post-treatment (Fig. 4, square symbols).

#### Longitudinal bilateral corticospinal tract cases (*n* = 15; all bone-marrow-derived mononuclear cells)

Pre- vs post-treatment repeated measures comparisons of RD changes across left and right CSTs reconstructed in each bilateral case (*n* = 15) showed no significant differences in magnitude of changes between left and right CST RD values (t(14) = 0.63, *P* |t| = 0.54). Interestingly, the average change in RD of the left CST was positive (mean = 0.0053 mm^2^/s), whereas the average change in RD of the left CST was negative (−0.0067 mm^2^/s) at 12 months post-treatment (Fig. 4, triangle, filled circle, and ‘x’ symbols). Note that RD drop is a marker of improved myelin while RD increase indicates poor myelin integrity.

In addition, a one-way analysis of variance (ANOVA) comparison of cases in participants <6 years (*n* = 7) and participants >6 years (*n* = 8) at baseline (prior to treatment infusion) showed no significant differences across age groups in changes in RD values in LH CST at 12 months post-treatment (F(1,14) = 0.08, *P* = 0.78) or RH CST (F(1,14) = 0.05, *P* = 0.94).

Owing to the observed differences in *directionality* of RD changes at 12 months post-treatment (some CSTs increased while others decreased), additional follow-up analyses were conducted (Fig. 4). Concordant changes in RD (both CSTs change in same direction) were observed in 10 of 15 cases (Fig. 4, filled circles and ‘x’ symbols). At 12 months post-treatment, RD decreased bilaterally in 6 of 15 cases (Fig. 4, filled circles; 3 left-handed, 1 right-handed, 2 unable to determine handedness). At 12 months post-treatment, RD increased bilaterally in 4 of 15 cases (Fig. 4, ‘x’ symbols; 2 left-handed, 1 right-handed, 1 unable to determine handedness). Discordant changes in RD (1 CST increased, other CST decreased) were observed in 5 of 15 cases (Fig. 4, triangles). At 12 months post-treatment, RD decreased in LH CST in 4 of 15 cases (3 left-handed, 1 unknown handedness). At 12 months post-treatment, RD decreased in RH CST in 1 of 15 cases (right-handed individual).

There was a significant improvement in RD averaged over the entire length of the reconstructed CST, which appears related to handedness. This suggests improvement in myelination of the CST, especially in the cord blood group.

#### Structural connectivity analysis

**Figure 5:**
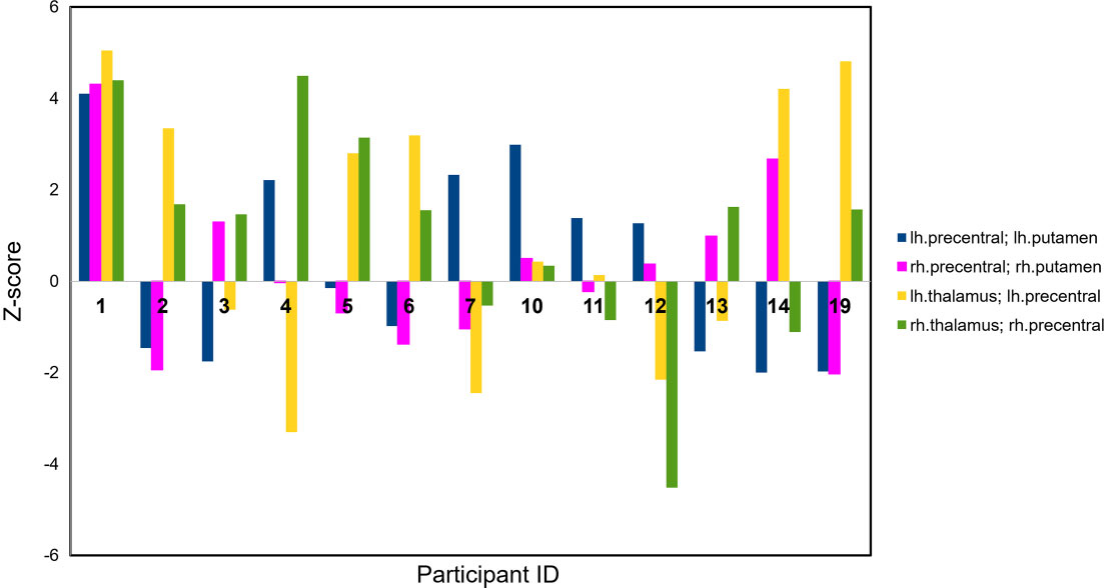
**Structural connectivity analyses.** Structural connectivity analyses based on fusion of anatomical and DTI MRI sequences were conducted on a subset (13/18) of participants where Freesurfer cortical parcellation was successfully completed. Positive *z*-scores reflect increased connectivity, and negative *z*-scores reflect decreased connectivity after treatment. Targeted connections (Freesurfer-based regions of interest) included the putamen-precentral and the thalamus-precentral. Each grouping of colored bars is an individual participant. CP = cerebral palsy, number corresponding to participant; lh = left hemisphere; rh = right hemisphere.

In addition to extracting microstructural metrics (RD) from reconstructed CST white matter pathways, we also conducted a structural connectivity analysis to evaluate changes in connectivity strength between grey matter regions, which are nodal components of cortico-striatal and thalamo-cortical motor pathways. As demonstrated in Fig. 5, connectivity strength increased in either or both of these pathways (corticio-striatal and thalamo-cortical) in each participant at 12 months post-treatment.

## Discussion

Our data demonstrate that the use of an autologous bone marrow or umbilical cord cellular therapy for children is safe and procedurally feasible in this patient population. Further, the data demonstrate striking concordance with the data from Sun *et al*.^8^ regarding GMFM-66 outcomes in this patient population. In terms of imaging, we demonstrated a potential treatment signal of reduced RD in at least 1 CST in 78% of 18 participants. Similar to previous studies,^8^ cell infusion at the doses used in our study did not dramatically alter motor function as captured by a change in GMFCS class (e.g. changing from a GMFCS-III to GMFCS-II).

The biological rationale for the use of these various cellular therapeutics has changed over time. While there was support in the 1990s for cell transplantation and engraftment using these types of cells to treat CNS injuries, the recognition of pleiotropic mechanisms of most of the cell types in modulating the immune response to injury has moved the field away from these mechanisms of action. Scepticism for using an adoptive cell therapy strategy in chronic injury persisted until Ramlackhansingh *et al*.’s work was published in 2011, where they described chronic microglial activation in patients up to 17 years after severe traumatic brain injury using PET imaging of upregulated translocator protein activity in microglia. Furthermore, the degree of activation (principally thalamic) was inversely related to neurocognitive function.^42^ This increased PET activity could represent a beneficial M2 microglial activation, but subsequent studies have supported the assertion that this signal indicates M1 or pro-inflammatory activity.^43^

Our previous work in traumatic brain injury and stroke has focused on the microglia as a therapeutic target, specifically in the acute period after injury.^12^ Thus, the idea that chronic activation was prominent in these settings rekindled our interest in this therapeutic strategy. Although some preclinical data and subsequent trials suggested treatment outside of the acute window was not beneficial,^44,45^ other data have suggested a potentially longer therapeutic window.^46^

### What constitutes clinically relevant or at least observable improvement?

Outcome tools used to measure responsiveness to therapy have been developed with an eye towards understanding the MCID after surgical treatment for CP.^47^ Furthermore, there are prognostic curves for the degree of expected clinical improvement in the GMFM-66 scores based upon patient age and their GMFCS level (I–V), as well as percentiles of performance within each level. In general terms, there is an ∼10–15 point difference between the GMFCS levels, and moving from 1 level to another would be a substantial and obvious gain.^28^ However, more modest gains have been described; in levels I–III, a 0.8–1.3 point improvement in the GMFM-66 constitutes a minimum clinically important improvement. To that end, our data suggest that there was a clinically important improvement in function using the MCID thresholds in *both* the placebo and treatment conditions of 2.2 and 1.5, respectively. These data should be interpreted with caution since the benchmarked MCID data to which we are comparing were derived from a cohort of less severely affected children; however, one would anticipate a lower change in the more severely affected cohort in our study. Similarly, 66% of the patients in the Duke trial were GMFCS level I–II. In that trial, there was an increase in the GMFM-66 of 1.7 for all treated patients and 2.2 for placebo.^8^ These data are strikingly similar to those reported in our study, and in both studies, the primary outcome of an improvement in the GMFM-66 scale relative to placebo controls was not achieved.

### Corticospinal tract imaging

In CP, associations between white matter integrity and motor function have been consistently reported. Of particular interest to our study is the growing literature reporting indices of white matter microstructural properties between lesioned versus non-lesioned CSTs within individual patients. These investigations are particularly informative for elucidating microstructural biomarkers, which can be non-invasively monitored over time even when standard measures of motor function are very difficult to assess/measure due to the severity of motor impairments exhibited by profoundly affected individuals. Numerous studies have reported elevated diffusivity metrics (MD, RD, AD) in lesioned CST when compared with non-lesioned CST within-participant,^48–53^ whereas FA failed to significantly differ between lesioned and non-lesioned CST. Therefore, we targeted our analyses to examine diffusivity, particularly RD, due to its consistent association with myelin integrity in translational studies.

The CST is organized bilaterally early in typical human development. As reviewed by Williams et al., the predictable developmental shift to a predominantly contralateral projection of CST from motor cortex to the spinal cord is largely driven by activity-dependent competition for synaptic connections with postsynaptic neuronal targets.^54^ In animal model studies, chemical inactivation of 1 motor cortex hemisphere early in development leads to altered reliance on ipsilateral CST projections later in development.^54–56^ Using transcranial magnetic stimulation, research studies of human infants with unilateral motor cortex injury have demonstrated altered CST development ipsi- and contralateral to the injured motor cortex, with uninjured CST contributions becoming more effective in activating targets ipsilaterally.^57^ In a recent DTI and transcranial magnetic stimulation study in 2 children with unilateral spastic CP, the child exhibiting evidence of ipsilateral CST innervation from the unaffected motor cortex to the affected hand demonstrated better functional scores than the child with evidence of preserved contralateral CST organization.^58^ This result suggests that atypical CST organization need not necessarily yield inferior functional performance. As expected, we mostly observed concordant bilateral changes in RD in 10 of 15 cases where each CST could be reconstructed in each hemisphere. In 60% of these cases (6/10), concordant decreases in bilateral CST RD occurred post-treatment. In addition, 100% of unilateral CST cases (3/3) exhibited decreased CST RD post-treatment. In our discordant cases (*n* = 5), directionality of changes in CST RD appeared to coincide with handedness. As reported by Krogsrud *et al*.,^59^ in children aged 4–11 years of age, CST RD typically decreases during this period of developmental maturation. Thus, our results corroborate this directionality of CST RD changes post-treatment, suggesting that treatment modulated the developmental trajectory of CST development in the appropriate direction and possibly reflects remodeling of myelination.

Our exploratory analyses of network-based structural connectivity pre- and post-treatment demonstrated highly variable patterns of change in connectivity strength in motor loops following treatment. Overall, these data are consistent with the concordant and discordant changes in RD observed in the CST reconstructions. Even though our study sample consisted of diverse clinical CP phenotypes, all 3 cases of unilateral CP treated with hUCB demonstrated decreased RD values in the preserved CST. Since none of our bilateral cases were treated with hUCB, we have insufficient information to determine whether treatment with hUCB differed from treatment with BMMNC. Whether changes in structural connectivity were mediated by modified efficiency or reconfiguration, this additional evidence of neural plasticity highlights the malleable nature of neural pathways in the developing brain.

### Dosing

At the time of the design of our trial, the dosing data from Sun *et al*.^8^ were not available. In their study, there was a potential dose-dependent treatment effect with doses over 2 × 10^7 ^cells/kg, with an improvement in the Peabody Developmental Motor Skills-Second Edition (PDMS-2) Gross Motor Quotient. The analysis of GMFM-66 12-month change score was not significantly different comparing the infused high dose versus placebo, but the actual minus expected GMFM-66 score was marginally improved in the placebo versus high dose group. Taken together, these data suggest that there may be a critical dose, or sequence of doses,^60^ required to generate an effect. There may be a dose–response relationship such that expanding a cord blood unit using various approaches could be considered. Our dosing was below the threshold of cells used in their study. Both our study and the Sun *et al*.^8^ trial demonstrate the importance of a randomized, blinded, placebo-controlled design, as both showed an MCID change in the placebo groups. These changes were of a magnitude such that an uncontrolled trial could attribute a clinically significant change to a proposed treatment.

### Study limitations

Our clinical trial has numerous limitations that are common to treating heterogeneous conditions and to cell-based therapies. First, the participants enrolled in this trial had a wide range of clinical and imaging findings that made pooling the small data sample problematic. For instance, global volumetric studies, as well as CST mapping studies, are problematic with hemispheric volume loss secondary to *in utero* stroke, and so on. Thus, we opted to present the data in multiple formats to account for these findings. Second, there is variability in cord blood doses that were available, and subsequent to the Duke trial,^8^ there were few families in the US with banked autologous units who had not already been treated. So, we were unable to enroll patients at the planned number. Thus, we analyzed data as all-cell-treated as well as separately. We recognize this limitation but also envision this as a pragmatic approach to evaluating a potential treatment signal in this population of patients. In addition, with our small sample size, our ability to compare hUCB and BMMNC head-to-head was limited. In addition, our small sample size and multiple design features further underpowered our trial, limiting our ability to to examine efficacy, and these results should be interpreted with caution. We believe that these imaging data are indicative of potential biological treatment effect/signal. To evaluate this further would ideally involve a larger cohort of patients, using higher doses with advanced imaging.

Our study suggests that both BMMNC and cord blood infusions are safe. There may be an improvement in CST diffusivity in some groups of patients that correlate with small improvements in the GMFCS scales. A larger autologous cord blood trial is impractical at current rates of autologous cord blood banking. Either increased private banking or matched units would be required to perform a larger-scale trial.

## Supplementary Material

fcac131_Supplementary_Data

## Abbreviations

AD =: axial diffusivity
ANOVA =: analysis of variance
BDDS =: Baseline dystonia disability scale
BDMS =: baseline dystonia movement scale
BMMNC =: bone-marrow-derived mononuclear cells
CBR =: Cord Blood Registry
CP =: cerebral palsy
CST =: corticospinal tract
dMRI =: diffusion MRI
DTI =: diffusion tensor imaging
EPI =: echo planar imaging
FA =: fractional anisotropy
FLAIR =: fluid-attenuated inversion recovery
GMFCS =: Gross Motor Classification System
GMFM =: Gross Motor Function Measure
GMFM-66 =: Gross Motor Function Measure-66 scale
GMFM-88 =: Gross Motor Function Measure-88 scale
hUCB =: human umbilical cord blood
IV =: intravenous
LH =: left hemisphere
MCID =: minimal clinically important difference
MD =: mean diffusivity
PDMS-2 =: Peabody Developmental Motor Skills-Second Edition
RD =: radial diffusivity
RH =: right hemisphere
sMRI =: structural MRI
SPGR =: spoiled gradient echo
SWAN =: susceptibility-weighted angiography
TE =: time to echo
TI =: inversion time
TNC =: total nucleated cell
TR =: repetition time
VABS-2 =: Vineland Adaptive Behavior Scales—Second Edition
v/v =: volume/volume
