# Supplementary material for: Autologous cellular therapy for cerebral palsy: a randomized, crossover trial
Source: Brain Commun. 2022 May 20;4(3):fcac131. doi: 10.1093/braincomms/fcac131 (PMC9188321; doi:10.1093/braincomms/fcac131)
Supplement: fcac131_Supplementary_Data [file fcac131_supplementary_data.docx]

**Supplementary Table 1.** Inclusion and Exclusion Criteria

| **Inclusion Criteria** |
| --- |
| 1. Children with diagnosis of Cerebral Palsy (spastic CP due to periventricular white matter damage or neonatal brain injury from perinatal stroke or intra-ventricular hemorrhage)  2. Gross Motor Function Classification Score Level II-V  3. 24 months to 10 years of age  4. English speaking, if verbal  5. Ability to travel to Houston, Texas for treatment and follow-up |
| **Exclusion Criteria** |
| 1. Know history of   - Intractable seizures - Traumatic brain injury - Genetic disorder (demonstrated by newborn screening or genetic diagnostic testing) - Recently treated or current infection - Renal insufficiency or altered renal function (as defined by serum creatinine > 1.5 mg/dL at screening) - Hepatic disease or altered liver function (as defined by serum glutamic pyruvic transaminase > 150 U/L [non-contusion related] and/or total bilirubin > 1.3 mg/dL at screening) - HIV positive (as demonstrated by positive blood test) - Immunosuppression (as defined by white blood cell count > 3,000 cells/mL at screening) - Infectious-related neurological injury - Sensitivity to ethylene oxide   2. If athetoid CP diagnosis, other etiologies such as degenerative, mitochondrial, and metabolic disorders must be excluded, and the outcome assessments must be conducted to assess for potential treatment effects  3. Normal brain magnetic resonance imaging  4. Evidence of acute illness at the time of infusion, such as, but not limited to, fever (temperature > 37.5ºC), vomiting, diarrhea, wheezing, or crackles  5. Progressing neurological disease (as defined by Batten disease, leukodystrophies, metabolic disorders, mitochondrial disorders, and/or neurotransmitter disorders)  6. Microcephaly, macrocephaly, cortical malformations, genetic disorders of dysgenesis brain malformations due to infection or metabolic disorders  7. Pulmonary disease requiring ventilator support  8. If hUCB candidate, banked blood cord cells totaling < 10 million/kg  9. If hUCB candidate, any positive maternal infectious disease test (hepatitis A/B, HIV-1, HIV-2, HTLV-1, HTLV-2, and/or syphilis  10. If hUCB candidate, cord blood sample contamination  11. Participation in a concurrent interventional research study  12. Unwillingness to return for follow-up visits  13. Contraindication to brain magnetic resonance imaging (e.g., metal implants, dental braces, etc.)  14. Any potential participant that the investigators feel, in their opinion the study intervention is unlikely to benefit; participant will be a screen failure  15. Previous participation in other stem cell treatment or research studies |

CP=cerebral palsy; hUCB= autologous umbilical cord blood; HTLV-1=human T-cell leukemia virus type 1; human T-cell leukemia virus type 2

**Supplementary Table 2.** Baseline Participant Characteristics

| **Variable** | **Baseline Summary Statistics** | | | |
| --- | --- | --- | --- | --- |
| ***Age*** | | | | |
| **Age, all treatment groups, years** | **2-4** | **5-7** | | **8-10** |
| All, n | 8 | 5 | | 7 |
| Male, n | 6 | 1 | | 5 |
| Female, n | 2 | 4 | | 2 |
| **Age by treatment group, years** | **BMMNC**  **(n=15)** | | **Cord Blood**  **(n=5)** | |
| Mean (SD) | 6.13 (2.92) | | 4 (2.12) | |
| Median | 6 | | 4 | |
| **Gestational Age, weeks** | **All Groups (n=20)** | **BMMNC (n=15)** | | **Cord Blood (n=5)** |
| Mean | 34.07 | 32.95 | | 37.4 |
| Median | 38 | 32 | | 38 |
| SD | 5.8 | 6.13 | | 3.13 |
| ***Behavioral and Motor Function Assessments*** | | | | |
| **Gross Motor Classification System for Cerebral Palsy (GMFCS), n (%)** | **All Groups (n=20)** | | | |
| Level 2 | 1 (5) | | | |
| Level 3 | 5 (25) | | | |
| Level 4 | 5 (25) | | | |
| Level 5 | 9 (45) | | | |
|  | **All Groups (n=20)** | **BMMNC (n=15)** | | **Cord Blood (n=5)** |
| **Baseline Dystonia Movement Scale (BDMS) Score** | | | | |
| Mean | 47.75 (31.68) | 48.81 (30.22) | | 45 (38.89) |
| Median | 52 | 51 | | 45 |
| **Baseline Dystonia Disability Scale (BDDS) Score** | | | | |
| Mean | 23.94 (7.82) | 24.77 (5.18) | | 21.8 (13.08) |
| Median | 26.5 | 25 | | 30 |
| ***Cerebral Palsy Classification*** | | | | |
| **Classification and Sub-type, n (%)** | **All Groups (n=20)** | **BMMNC (n=15)** | | **Cord Blood (n=5)** |
| Spastic | 12 (60) | 7 (46.7) | | 5 (100) |
| Unilateral | 3 (15) | 1 (6.7) | | 2 (40) |
| Bilateral | 9 (45) | 6 (40) | | 3 (60) |
|  |  |  | |  |
| Dyskinetic | 8 (40) | 8 (53.3) | | 0 (0) |
| Dystonic | 5 (25) | 5 (33.3) | | 0 (0) |
| Choreo-athetotic | 3 (15) | 3 (20) | | 0 (0) |
| Ataxic | 0 (0) | 0 (0) | | 0 (0) |

**Supplementary Table 3.** Adverse Events.

| **Participant ID** | **Adverse Event** | **Stem Cell Group** | **Relationship to Stem Cell Intervention** |
| --- | --- | --- | --- |
| 1 | Nose Bleed Following Flight Home | BMMNC | Unrelated |
| 2 | Upper Respiratory Infection  Gastroenteritis | BMMNC | Unrelated  Unrelated |
| 3 | Upper Respiratory Infection | BMMNC | Unrelated |
| 4 | Mild Respiratory Infection  Mild Respiratory Infection | BMMNC | Unrelated  Unrelated |
| 4 | Upper Respiratory Infection | BMMNC | Unrelated |
| 9 | Upper Respiratory Infection  Gastroenteritis  Gastroenteritis | BMMNC | Unrelated  Unrelated  Unrelated |
| 10 | Ear Infection | BMMNC | Unrelated |
| 11 | Pneumonia  Increase in Seizure Activity (History of Seizure Disorder) | BMMNC | Unrelated  Unrelated |
| 13 | Vitiligo | BMMNC | Unrelated |
| 14 | Ear Infection  Nasal Congestion with Fever | BMMNC | Unrelated  Unrelated |
| 17 | Febrile Seizure (History of Febrile Seizures) | hUBC | Unrelated |
| 18 | Bloody Stools/Flair-up of Chrohn’s Disease  Increase in Seizure Activity (History of Seizure Disorder)  Planned Left Hip Osteotomy  Postoperative Upper Respiratory Infection (After Hip Surgery) | hUBC | Unrelated  Unrelated  Unrelated  Unrelated |
| 19 | Upper Respiratory Infection | hUBC | Unrelated |
| 20 | Nasal Congestion (History of Sinus Allergies)  Pneumonia/History of Upper Respiratory Infections  Increase in Seizure Activity (History of Seizure Disorder)  Croup/Pneumonia | hUBC | Unrelated  Unrelated  Unrelated  Unrelated |
